# Efficacy of structured spiritual care education program on nurses’ spiritual well-being and clinical competence: a randomized controlled trial

**DOI:** 10.1186/s12912-026-04987-0

**Published:** 2026-07-03

**Authors:** Mehrvash Hemati, Azita Jaberi, Naval Heydari, Marzieh Momennasab

**Affiliations:** 1 Department of Nursing, Community Based Psychiatric Care Research Center, School of Nursing and Midwifery, Shiraz University of Medical Sciences, Shiraz, Iran; 2 Department of Nursing, School of Nursing and Midwifery, Shiraz University of Medical Sciences, Shiraz, Iran

**Keywords:** Spiritual care, Nursing education, Clinical competence, Spiritual well-being

## Abstract

**Background:**

Spiritual care is a key component of holistic nursing practice, yet many nurses feel insufficiently prepared to provide it effectively. This study aimed to evaluate the effect of a structured spiritual care education on nurses’ spiritual well-being and clinical competence.

**Methods:**

This randomized controlled trial was conducted on 120 female nurses working in a specialized hospital in Iran from March 2024 to January 2025. Participants were randomly assigned in a 1:1 ratio to the intervention (*n* = 60) and control (*n* = 60) groups using block randomization with a block size of four and allocation concealment via sequentially numbered, opaque, sealed envelopes. The intervention group received a four-session structured spiritual care education program based on interactive lectures, scenario-based learning, and reflective discussions using the Gibbs reflective cycle, while the control group received routine training. Outcomes included spiritual well-being (measured by the Spiritual Well-Being Scale) and clinical competence (measured by the Clinical Competence Questionnaire), assessed at baseline, immediately post-intervention, and at two-month follow-up.

**Results:**

After the intervention, the intervention group showed significantly higher spiritual well-being scores compared to the control group, including both religious and existential dimensions (*p* < 0.001). Clinical competence scores were also significantly higher in the intervention group across most domains, including clinical care, interpersonal relationships, legal–ethical practice, professional development, and teaching–coaching (all *p* < 0.001). These improvements were sustained at the two-month follow-up. Effect sizes ranged from small to large, and mixed repeated-measures analysis confirmed a significant group × time interaction (*p* < 0.001), indicating greater improvement in the intervention group over time.

**Conclusion:**

Structured scenario-based spiritual care education can effectively improve nurses’ spiritual well-being and clinical competence. We recommend integrating such programs into nursing education and continuing professional development.

**Trial registration:**

Retrospectively registered in the Iranian Registry of Clinical Trials (IRCTID:**IRCT20220927056047N2**; https://en.irct.ir/trial/89352) on 2026–05-02.

## Background

Spiritual care is increasingly recognized as a fundamental component of holistic nursing practice [1]. International frameworks, including those of the World Health Organization, conceptualize health as a multidimensional construct that extends beyond physical and psychological domains to include spiritual well-being [2]. Spirituality contributes to individuals’ sense of meaning, purpose, and hope, particularly in the context of illness, suffering, and end-of-life experiences [3]. As frontline providers of care, nurses are uniquely positioned to assess and address patients’ spiritual needs through therapeutic presence, empathetic communication, and supportive interventions [4]. Addressing these needs has been associated with improved psychological adjustment, reduced anxiety and depression, enhanced coping, and greater satisfaction with care, as well as preservation of dignity and better adaptation to illness [5–7]. Despite this recognized importance, many nurses report insufficient preparation and lack of confidence in delivering spiritual care in clinical settings [8].

Spiritual care education programs are structured interventions aimed at improving nurses’ ability to integrate patients’ spiritual needs into clinical practice [9]. Spiritual well-being reflects an individual’s perceived meaning, purpose, and inner peace derived from personal beliefs and existential understanding [10], while clinical competence refers to the integration of knowledge, skills, and professional attitudes required to deliver safe and holistic nursing care [11]. These constructs are interrelated, as enhancing nurses’ competencies in spiritual care may contribute not only to improved clinical performance but also to their own spiritual well-being and professional development [12, 13].

In specialized maternal and neonatal care settings, nurses provide continuous care to women and newborns experiencing physically and emotionally complex conditions, including childbirth, neonatal complications, and clinical emergencies [14]. These high-acuity environments are characterized by time pressure, high workload, and frequent exposure to emotionally distressing situations, which may restrict opportunities to address patients’ spiritual needs within routine care [15, 16]. This highlights the importance of structured interventions that can support nurses in integrating spiritual care into everyday clinical practice [17].

Beyond patient outcomes, nurses’ own spiritual well-being has gained increasing attention as a determinant of both personal and professional functioning [18]. Spiritual well-being, reflecting a sense of inner coherence and connectedness, has been linked to lower levels of burnout, greater resilience, and higher job satisfaction [19, 20]. Moreover, nurses with higher spiritual well-being are more likely to establish therapeutic relationships, demonstrate empathy, and respond effectively to patients’ spiritual concerns [21, 22]. However, spiritual care remains insufficiently integrated into nursing education, with limited structured, evidence-based training programs and underexplored in nursing research [23, 24].

Alongside nurses’ spiritual well-being, clinical competence is a fundamental component of high-quality nursing care and is essential for the delivery of safe, effective, and holistic practice [25]. Despite its importance, evidence suggests that nurses’ competence in delivering comprehensive care, including broader dimensions of patient-centered and holistic nursing practice, is suboptimal in many clinical settings [12]. Previous research has identified persistent gaps in nurses’ knowledge, skills, and confidence in providing holistic care, which may compromise the quality and completeness of patient care [26]. These shortcomings are largely attributed to limited integration of spiritual care content within nursing education, insufficient practical training opportunities, and healthcare systems that prioritize technical and task-oriented aspects of care [27, 28]. As a result, the consistent incorporation of holistic and spiritually sensitive care into routine nursing practice remains challenging, highlighting the need for structured, evidence-based educational interventions aimed at strengthening nurses’ clinical competence [29].

Educational interventions represent an effective strategy to enhance nurses’ competence in delivering holistic and person-centered care, including spiritual dimensions [12, 30]. Previous studies have shown that structured spiritual care education can improve nurses’ knowledge, attitudes, and clinical skills [26]. However, most existing programs rely predominantly on traditional didactic approaches, with limited use of interactive, reflective, and scenario-based strategies that align with competence-based nursing education [26, 27]. In addition, the majority of prior research has primarily focused on patient-related outcomes or nurses’ spiritual care competence, while the potential impact of such interventions on nurses’ own spiritual well-being remains underexplored [26, 31]. Importantly, limited studies have simultaneously evaluated both professional competence and personal spiritual well-being within rigorous randomized controlled trial designs with adequate sample sizes and follow-up assessments [12, 26].

A growing body of research has examined the effects of spiritual care education on nurses’ outcomes, generally reporting positive effects on spiritual well-being and clinical competence outcomes [12, 13, 26]. However, much of this evidence is derived from quasi-experimental studies with relatively small sample sizes and limited or no follow-up assessments, which may restrict the strength and generalizability of findings. In addition, although studies conducted in Iran have reported beneficial effects of spiritual care education on outcomes such as empathy and professional commitment [32], limited research has simultaneously evaluated both nurses’ spiritual well-being and clinical competence within a randomized controlled trial design.

Therefore, this study aimed to evaluate the effectiveness of a structured, scenario-based spiritual care education program on nurses’ spiritual well-being and clinical competence. A randomized controlled trial design with a two-month follow-up assessment was used to address existing gaps in the literature. It was hypothesized that nurses in the intervention group would demonstrate significantly higher levels of both spiritual well-being and clinical competence compared with those in the control group receiving routine nursing training.

## Methods

### Design and participants

This two-group randomized controlled trial with a pretest–posttest design was conducted among 120 nurses employed at Shooshtari Hospital, a specialized mother and child hospital affiliated with Shiraz University of Medical Sciences in Shiraz, Iran. Because the hospital exclusively provides healthcare services for women, all participants in this study were female nurses. The study was conducted from March 2024 to January 2025. Based on the experimental study by Abusafia et al. [26], which evaluated changes in nurses’ competence before and after a spiritual care educational intervention, the reported post-intervention mean (μ) and standard deviation (SD) was 105.89 (7.46) in the intervention group and 101.18 (7.17) in the control group. Using these values, the sample size was calculated with G-Power software, assuming a significance level (α) of 0.05 and a statistical power (1 – β) of 0.80. The estimated minimum sample size was 52 participants per group. To account for an anticipated attrition rate of 15%, the final sample size was adjusted to 60 participants per group.

The inclusion criteria were willingness to participate in the study, holding at least a bachelor’s degree in nursing, having at least one year of clinical experience, and being actively employed in clinical wards during the study period. The exclusion criteria included withdrawal from the study at any stage, absence from one or more intervention sessions, failure to complete the study questionnaires at the pretest or posttest stages, and participation in other spirituality-related programs during the study period.

**Fig. 1:**
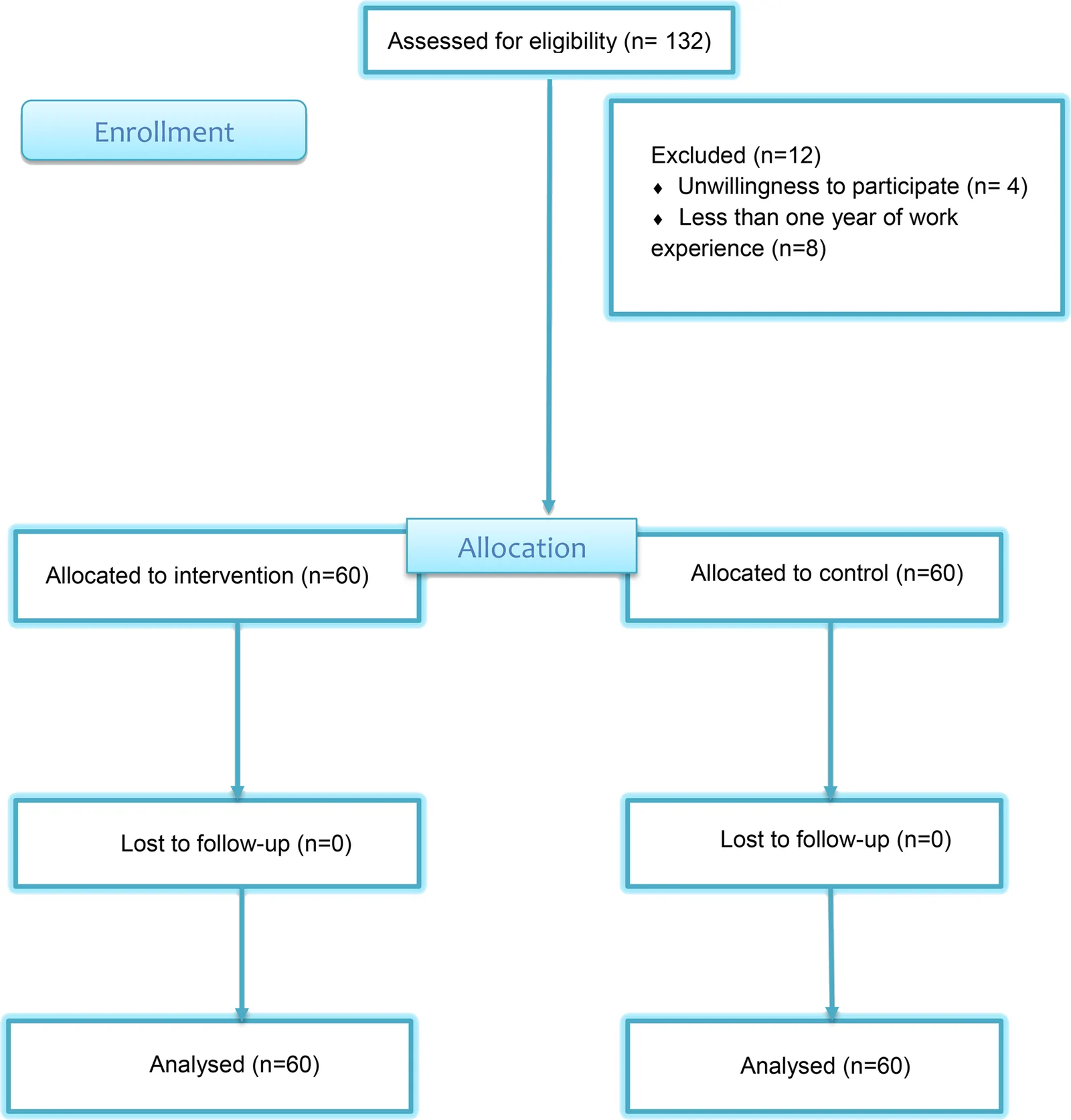
CONSORT flow diagram of participant recruitment, allocation, follow-up, and analysis

Eligibility was determined based on the predefined inclusion and exclusion criteria. After all eligible participants had been recruited, they were randomly assigned in a 1:1 ratio to either the intervention group (*n* = 60) or the control group (*n* = 60) using block randomization with a block size of four generated by Random Allocation Software (version 2.0). The allocation sequence was concealed in sequentially numbered, opaque, sealed envelopes, which were opened by a research assistant at the time of assignment. Following randomization, participants in the control group completed both baseline and follow-up assessments before the educational intervention was delivered to participants in the intervention group. Subsequently, baseline assessments were conducted for the intervention group, followed by implementation of the educational program and post-intervention assessments. This approach was adopted to minimize contamination and information exchange between groups. Furthermore, outcome data were collected by a separate research assistant who was blinded to group allocation, ensuring unbiased data collection. This study was reported in accordance with the CONSORT guidelines. The participant enrollment, allocation, follow-up, and analysis are illustrated in the CONSORT flow diagram (Fig. 1).

### Intervention

The intervention consisted of a spiritual care educational workshop conducted at the Spiritual Care Center of Shooshtari Hospital. The workshop was facilitated by two nursing faculty members with expertise in spiritual care and spirituality education in nursing. Both facilitators had completed formal training courses in spiritual care and possessed extensive experience in teaching, research, and scholarly publications, including books and research projects related to spirituality and spiritual care in nursing.

The program was delivered over four weekly sessions during a one-month period, with each session lasting approximately 3–4 hours. Each session began with an interactive lecture introducing key concepts related to spirituality, spiritual health, and spiritual care in nursing practice. This was followed by interactive learning activities including small-group discussions, analysis of clinical scenarios, and guided reflective dialogue.

Participants were divided into small groups of approximately five nurses to discuss clinical scenarios designed to stimulate reflection on the spiritual dimensions of patient care. The scenarios were organized progressively, beginning with basic spiritual needs and gradually moving toward more complex clinical situations requiring therapeutic presence and integration of spiritual care into routine nursing practice.

The educational content and clinical scenarios were developed based on a review of the international literature on spiritual care in nursing, relevant textbooks, clinical practice resources, and the researchers’ educational and clinical experience in the field of spirituality and spiritual care. The content validity of the educational materials was assessed and confirmed by five faculty members from the school of nursing.

Reflective discussions were structured using the Gibbs reflective cycle, which includes six stages: description, feelings, evaluation, analysis, conclusion, and action plan. Guided reflective questions corresponding to each stage were used to facilitate discussion. During the sessions, participants analyzed the presented scenarios, shared their perspectives and emotional responses, and discussed possible nursing interventions to address patients’ spiritual needs.

Intervention fidelity was ensured through the use of standardized session protocols and delivery of all sessions by the same facilitators. Participant attendance was monitored throughout the program to ensure full exposure to the intervention. Participants who attended all four sessions were considered to have completed the intervention. At the end of each session, facilitators summarized the key learning points and addressed participants’ questions.

The control group did not receive the structured educational intervention and continued with the routine nursing education and clinical training available in the hospital. For ethical considerations, the educational materials were provided to the control group after completion of the post-test assessment. The structure and educational content of the educational sessions are presented in Table 1.

**Table 1:** Structure and educational content of the spiritual care education program

Session	Content	Teaching methods	Reflective scenarios
Session 1	Introduction to spirituality, spiritual health, and spiritual care in nursing; importance of addressing patients’ spiritual needs; overview of reflective learning and the Gibbs reflective cycle	Interactive lecture; facilitated discussion; introduction to small-group reflection	Scenario 1 – Prayer and worship (patients’ religious practices)
Session 2	Recognition of patients’ spiritual needs and appropriate supportive nursing responses	Interactive lecture; small-group reflective discussion using the Gibbs reflective cycle; guided reflective questions; facilitator feedback	Scenario 2 – Instilling hope (hope and emotional support); Scenario 3 – Connection with nature (sense of connectedness)
Session 3	Therapeutic presence and communication in spiritual care; responding to patients’ search for meaning and purpose	Interactive lecture; reflective group discussion; experience sharing; scenario analysis	Scenario 4 – Therapeutic presence; Scenario 5 – Search for meaning and purpose
Session 4	Integration of spiritual care into routine nursing practice; ethical and cultural considerations in spiritual care	Interactive lecture; reflective group discussion; scenario analysis; action planning; summary of key learning points	Scenario 6 – Forgiveness and reconciliation; Scenario 7 – Religious support and presence of a religious figure

### Data collection

Data were collected using three instruments: a demographic questionnaire, the Clinical Competence Questionnaire for Registered Nurses (CIRN), and the Spiritual Well-Being Scale (SWBS). Assessments were conducted at three time points: baseline, post-intervention, and two-month follow-up. Participants in both the intervention and control groups completed the questionnaires at all three time points.

### Demographic questionnaire

The demographic questionnaire collected information on participants’ age, years of work experience, marital status, educational level, number of children, clinical unit type, employment status, work shift pattern, and prior participation in spiritual care workshops.

### Clinical Competence Questionnaire for Registered Nurses (CIRN)

Clinical competence was assessed using the Clinical Competence Questionnaire for Registered Nurses (CIRN) developed by Liu et al. in 2007. The instrument includes 55 items across seven domains: clinical care, leadership, interpersonal relationships, legal–ethical practice, professional development, teaching–coaching, and research aptitude–critical thinking. Items are rated on a 5-point Likert scale (0–4), with total scores ranging from 0 to 220, where higher scores indicate greater competence [33]. The CIRN was used in this study as a self-report measure of nurses’ perceived clinical competence. The original version demonstrated good psychometric properties, including acceptable content validity (CVI = 0.85), internal consistency (Cronbach’s alpha = 0.89 for the total scale), and test–retest reliability (*r* = 0.76–0.91). Construct validity was supported by a seven-factor structure explaining 53.92% of the variance. Criterion and discriminant validity were supported [33]. The Persian version showed excellent validity and reliability, with strong model fit in confirmatory factor analysis (CFI = 0.98, RMSEA = 0.063), high internal consistency (Cronbach’s alpha = 0.967), and strong stability (ICC = 0.939) [34]. In the present study, the Cronbach’s alpha coefficient for the instrument was 0.959, indicating excellent internal consistency.

### Spiritual Well-Being Scale (SWBS)

Spiritual well-being was measured using the Spiritual Well-Being Scale (SWBS) developed by Paloutzian and Ellison in 1982 [35]. The scale consists of 20 items covering two dimensions: religious well-being and existential well-being. Items are rated on a 6-point Likert scale (1–6), with total scores ranging from 20 to 120, where higher scores indicate greater spiritual well-being. Scores are categorized as low (20–40), moderate (41–99), and high (100–120). The SWBS has demonstrated strong psychometric properties, including good internal consistency (α = 0.83–0.94), high test–retest reliability (0.86–0.96), and confirmed two-factor structure across studies [36, 37]. The Persian version also showed acceptable validity (CVI > 0.80), satisfactory model fit (CFI ≈ 0.92, RMSEA ≈0.08), good internal consistency (α = 0.84–0.92), and strong stability (ICC = 0.825) [38]. In the present study, the Cronbach’s alpha coefficient for the instrument was 0.930, indicating excellent internal consistency.

### Data analysis

Data were analyzed using SPSS software, version 27.0 (IBM Corp., Armonk, NY, USA). The normality of continuous variables was assessed using the Shapiro–Wilk test. Categorical variables were compared using the Chi-square test or Fisher’s exact test, as appropriate. Between-group comparisons were performed using independent samples t-tests or the Mann–Whitney U test, depending on data distribution. Within-group changes over time were analyzed using repeated-measures ANOVA for normally distributed variables and the Friedman test for non-normally distributed variables. In addition, a mixed repeated-measures ANOVA was conducted to examine the effects of time, group, and their interaction on study outcomes. Where the assumption of sphericity was violated, Greenhouse–Geisser corrections were applied. Cohen’s d effect size with 95% confidence intervals was calculated as the difference in mean scores between the intervention and control groups divided by the pooled standard deviation at each time point, to quantify the magnitude of between-group differences and facilitate interpretation of effect size, interpreted as small (0.2), medium (0.5), and large (0.8) [39]. A *p*-value < 0.05 was considered statistically significant.

## Results

Among the 120 participants enrolled in the study, all completed the intervention and follow-up assessments. The median age (Q1–Q3) was 38.0 (29.0–42.0) years in the intervention group and 36.0 (31.2–44.0) years in the control group (*p* = 0.508). Similarly, the median years of work experience were 14.0 (4.0–18.0) in the intervention group and 9.5 (4.2–17.7) in the control group (*p* = 0.605). There were no statistically significant differences between the two groups in baseline demographic characteristics, indicating baseline comparability between groups (Table 2).

**Table 2:** Baseline demographic characteristics of participants

Variables		Total (*n* = 120)	Intervention (*n* = 60)	Control (*n* = 60)	*p*-value
		n (%)	n (%)	n (%)	
Marital status	Single	37 (30.8)	16 (26.7)	21 (35.0)	0.323*
	Married	83 (69.2)	44 (73.3)	39 (65.0)	
Number of children	0	53 (44.2)	23 (38.3)	30 (50.0)	0.198*
	1	28 (23.3)	18 (30.0)	10 (16.7)	
	≥2	39 (32.5)	19 (31.7)	20 (33.3)	
Educational degree	Bachelor’s degree	109 (90.8)	53 (88.3)	56 (93.3)	0.343*
	Master’s degree	11 (9.2)	7 (11.7)	4 (6.7)	
Work shift pattern	Morning	31 (25.8)	15 (25.0)	16 (26.7)	0.167**
	Evening	3 (2.5)	3 (5.0)	0 (0.0)	
	Night	8 (6.7)	6 (10.0)	2 (3.3)	
	Rotating	78 (65.0)	36 (60.0)	42 (70.0)	
Employment status	Official	76 (63.3)	36 (60.0)	40 (66.7)	0.449*
	Non-Official***	44 (36.7)	24 (40.0)	20 (33.3)	
prior spiritual care workshop participation	Yes	5 (4.2)	3 (5.0)	2 (3.3)	0.990**
	No	115 (95.8)	57 (95.0)	58 (96.7)	
Clinical unit type	Labor	12 (10.0)	6 (10.0)	6 (10.0)	0.843*
	Operating Room	12 (10.0)	7 (11.7)	5 (8.3)	
	Obstetrics	22 (18.3)	9 (15.0)	13 (21.7)	
	Neonatal Intensive Care Unit	29 (24.2)	13 (21.7)	16 (26.7)	
	Emergency Room	28 (23.3)	15 (25.0)	13 (21.7)	
	Outpatient Department	17 (14.2)	10 (16.7)	7 (11.7)	

* P: Pearson Chi-square test

** P: Fisher’s exact test

***Project-based, long-term contractual, short-term contractual, partnership

At baseline, no significant differences were observed between the intervention and control groups in the total spiritual well-being score or in the religious and existential dimensions (all *p* > 0.05). Significant improvements over time were observed in the intervention group for total spiritual well-being and for both the religious and existential dimensions following the educational intervention (within-group *p* ≤ 0.001). In contrast, the control group showed either no significant changes or smaller changes over time across the study outcomes. Furthermore, at both the immediate post-intervention assessment and the two-month follow-up, the intervention group demonstrated significantly higher scores in total spiritual well-being and in both the religious and existential dimensions compared with the control group (all *p* < 0.05). Effect size analyses demonstrated small to large between-group differences according to Cohen’s d (0.367–2.116) (Table 3).

**Table 3:** Comparison of spiritual well-being scores between the intervention and control groups at baseline, post-intervention, and two-month follow-up

Spiritual well-being scale	Evaluation time	Group		*p*-value (between-groups)	Cohen’s d (95% CI)
		Intervention n = 60	Control n = 60		
		Mean ± SD Median (Q1–Q3)	Mean ± SD Median (Q1–Q3)		
Religious	Baseline	41.2 ± 9.4 41.0 (34.0–46.0)	41.6 ± 9.3 40.0 (36.0–50.0)	0.815*	−0.043 (−0.401, 0.315)
	Post-intervention	46.0 ± 6.4 44.5 (41.2–50.7)	41.8 ± 8.3 40.0 (36.0–49.7)	0.002**	0.568 (0.202, 0.932)
	Two-month follow-up	44.7 ± 6.3 44.0 (40.0–49.5)	41.9 ± 8.9 40.0 (36.2–50.0)	0.034**	0.367 (0.005, 0.727)
p-value (within-groups)		<0.001***	0.809***		
Existential	Baseline	32.9 ± 9.5 33.0 (26.0–38.5)	34.1 ± 5.4 33.5 (31.0–38.0)	0.378*	−0.162 (−0.520, 0.197)
	Post-intervention	45.1 ± 5.3 45.5 (42.2–48.0)	34.7 ± 4.6 35.0 (31.0–39.0)	<0.001**	2.116 (1.665, 2.561)
	Two-month follow-up	42.7 ± 5.3 42.5 (39.0–46.0)	35.1 ± 4.8 35.0 (32.0–38.7)	<0.001*	1.512 (1.103, 1.916)
p-value (within-groups)		<0.001***	<0.001***		
Total	Baseline	74.1 ± 17.5 71.5 (61.2–86.5)	75.7 ± 13.8 73.0 (67.7–87.5)	0.568*	−0.105 (−0.462, 0.254)
	Post-intervention	91.1 ± 10.6 90.5 (84.0–99.0)	76.4 ± 12.1 74.0 (69.2–86.7)	<0.001*	1.294 (0.898, 1.686)
	Two-month follow-up	87.4 ± 10.7 86.5 (79.0–94.7)	76.9 ± 12.8 74.5 (69.2–88.2)	<0.001*	0.886 (0.509, 1.259)
p-value (within-groups)		0.001****	<0.002****		

* P: Independent samples t-test

** P: Mann–Whitney U test

*** P: Friedman test

**** P: Repeated Measures ANOVA

**Fig. 2:**
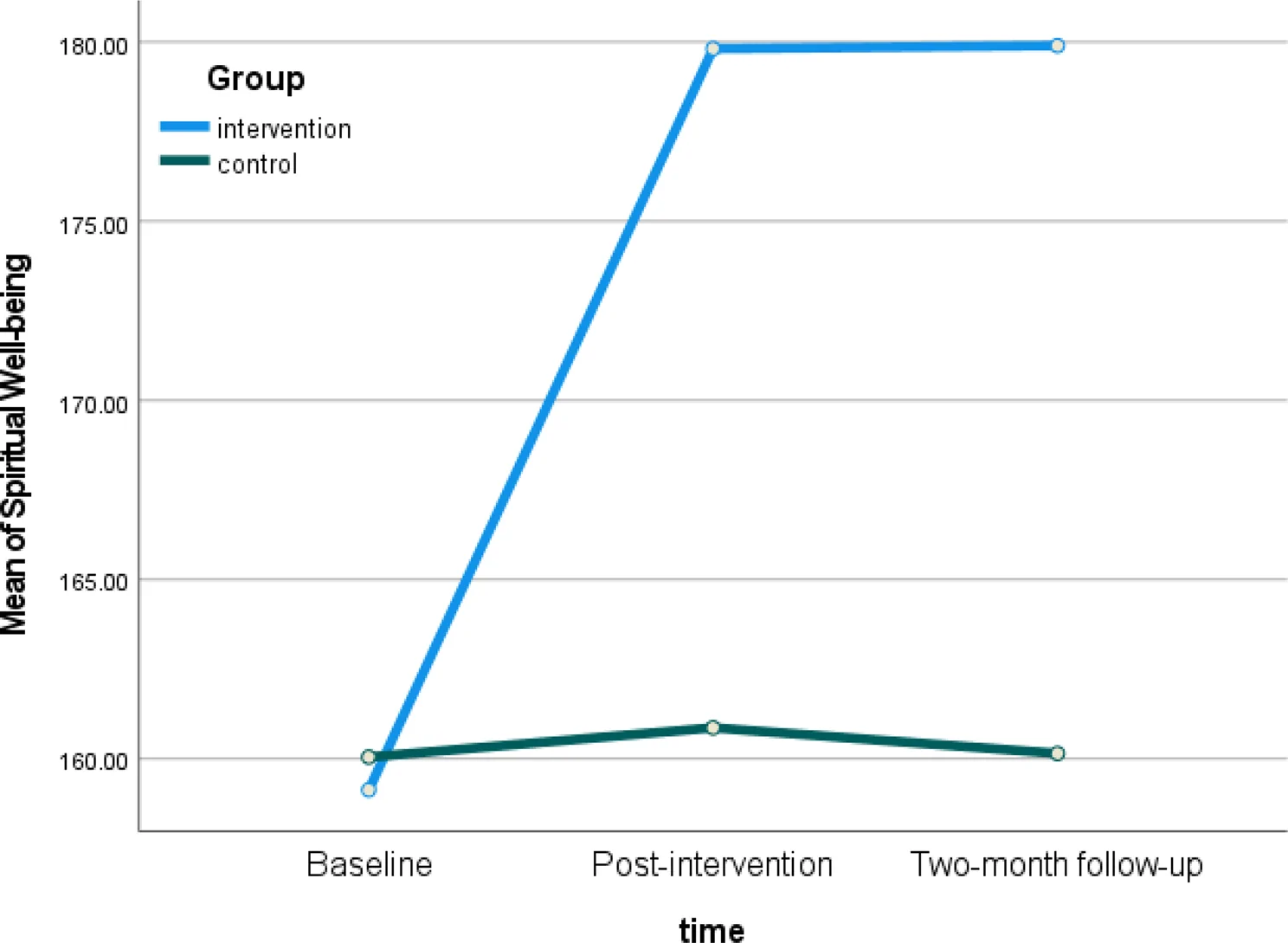
Mean spiritual well-being scores across three time points (baseline, post-intervention, and two-month follow-up) in the intervention and control groups

Because the assumption of sphericity was violated, Greenhouse–Geisser corrections were applied. Mixed repeated-measures ANOVA demonstrated significant main effects of time and significant time × group interactions for total spiritual well-being and for both the religious and existential dimensions (all *p* < 0.001). Significant main effects of group were observed for the total spiritual well-being score and the existential dimension, whereas the group effect for the religious dimension was not statistically significant (*p* = 0.134). These findings indicate that changes in spiritual well-being over time differed significantly between the intervention and control groups, with greater improvements observed among participants in the intervention group (Table 4). The trends in spiritual well-being scores across the three assessment time points are presented in Fig. 2.

**Table 4:** Mixed repeated measures ANOVA results for spiritual well-being total score and subscales

Outcome	Effect	F (df)	*p*-value	Partial η^2^
Total score	Time	398.9 (1.4, 160.6)	<0.001	0.772
	Group	13.2 (1,118)	<0.001	0.100
	Time × Group	365.3 (1.4, 160.6)	<0.001	0.756
Religious	Time	48.5 (1.4,160.6)	<0.001	0.291
	Group	2.3 (1,118)	0.134	0.019
	Time × Group	40.9 (1.4,160.6)	<0.001	0.257
Existential	Time	162.4 (1.2,144.7)	<0.001	0.579
	Group	30.7 (1,118)	<0.001	0.206
	Time × Group	129.7 (1.2,144.7)	<0.001	0.524

At baseline, no significant differences were observed between the intervention and control groups in the total clinical competence score or any of its seven dimensions (all *p* > 0.05). Following the intervention, the intervention group demonstrated significant improvements in several dimensions of clinical competence, including clinical care, interpersonal relationships, legal–ethical practice, professional development, and teaching–coaching (within-group *p* ≤ 0.001). In contrast, the control group showed no statistically significant changes across most dimensions over time. Between-group comparisons revealed significantly higher scores in the intervention group for clinical care, interpersonal relationships, legal–ethical practice, professional development, teaching–coaching, and the total clinical competence score at both post-intervention and two-month follow-up assessments (all *p* < 0.05). No significant between-group differences were observed for the leadership or research aptitude–critical thinking dimensions at either post-intervention or follow-up assessments (*p* > 0.05). Effect size analyses demonstrated small to large between-group differences, with the largest effects observed for clinical care and interpersonal relationships (Table 5).

**Table 5:** Comparison of clinical competence scores between the intervention and control groups at baseline, post-intervention, and two-month follow-up

Clinical competence scale	Evaluation time	Group		p-value (between-groups)	Cohen’s d (95% CI)
		Intervention n = 60	Control n = 60		
		Mean ± SD Median (Q1–Q3)	Mean ± SD Median (Q1–Q3)		
Clinical Care	Baseline	27.8 ± 4.1 27.5 (25.0–31.0)	28.0 ± 4.0 28.0 (25.0–31.0)	0.857*	−0.033 (−0.391, 0.325)
	Post-intervention	33.3 ± 3.5 34.0 (30.0–36.0)	28.2 ± 3.7 28.0 (25.0–31.0)	<0.001**	1.433 (1.029, 1.833)
	Two-month follow-up	34.2 ± 3.2 35.0 (32.0–36.7)	27.9 ± 3.9 28.0 (25.0–31.0)	<0.001*	1.788 (1.351, 2.199)
p-value (within-groups)		<0.001***	0.002****		
Leadership	Baseline	26.9 ± 4.5 26.0 (24.0–31.0)	27.0 ± 4.4 26.0 (24.0–31.0)	0.874**	−0.034 (−0.392, 0.324)
	Post-intervention	28.6 ± 4.4 28.0 (26.0–32.0)	27.1 ± 4.3 26.0 (24.0–31.0)	0.053**	0.340 (−0.021, 0.700)
	Two-month follow-up	28.1 ± 4.4 27.0 (25.2–31.7)	27.2 ± 4.1 26.5 (24.0–31.0)	0.252**	0.208 (−0.151, 0.566)
p-value (within-groups)		<0.001***	0.375***		
Interpersonal Relationships	Baseline	22.2 ± 2.8 22.0 (20.0–24.0)	22.3 ± 2.8 22.0 (21.0–24.0)	0.706**	−0.047 (−0.405, 0.311)
	Post-intervention	28.4 ± 2.0 29.0 (27.2–29.0)	22.5 ± 2.7 22.0 (21.0–24.0)	<0.001**	2.486 (2.005, 2.961)
	Two-month follow-up	29.4 ± 2.0 29.5 (28.0–31.0)	22.2 ± 2.6 22.0 (21.0–24.0)	<0.001**	3.049 (2.518, 3.574)
p-value (within-groups)		<0.001***	0.071***		
Legal and Ethical Practice	Baseline	25.1 ± 3.9 26.0 (23.0–28.0)	25.2 ± 3.8 26.0 (23.0–28.0)	0.851**	−0.039 (−0.397, 0.319)
	Post-intervention	27.4 ± 3.1 28.0 (26.0–30.0)	25.3 ± 3.7 26.0 (23.0–28.0)	<0.001**	0.617 (0.249, 0.982)
	Two-month follow-up	27.4 ± 3.2 28.0 (26.0–30.0)	25.5 ± 3.5 26.0 (23.0–28.0)	0.002**	0.576 (0.209, 0.940)
p-value (within-groups)		<0.001***	0.009***		
Professional Development	Baseline	17.4 ± 3.0 17.0 (15.2–19.0)	17.5 ± 2.8 17.5 (16.0–19.0)	0.731**	−0.050 (−0.408, 0.308)
	Post-intervention	19.3 ± 2.6 20.0 (18.0–21.0)	17.6 ± 2.8 17.5 (16.0–19.0)	<0.001**	0.618 (0.250, 0.983)
	Two-month follow-up	18.7 ± 2.7 18.5 (17.0–20.0)	17.3 ± 2.7 17.0 (16.0–19.0)	0.002**	0.508 (0.144, 0.871)
p-value (within-groups)		<0.001***	<0.001***		
Teaching–Coaching	Baseline	17.3 ± 2.9 17.0 (16.0–19.0)	17.4 ± 2.8 17.0 (16.0–19.0)	0.880 **	−0.035 (−0.393, 0.323)
	Post-intervention	19.1 ± 2.6 19.0 (18.0–21.0)	17.5 ± 2.9 17.0 (16.0–19.0)	<0.001**	0.595 (0.228, 0.959)
	Two-month follow-up	18.6 ± 2.6 18.0 (17.0–21.0)	17.7 ± 2.5 17.0 (16.0–19.0)	0.022**	0.366 (0.004, 0.726)
p-value (within-groups)		<0.001***	<0.003***		
Research Aptitude and Critical Thinking	Baseline	22.4 ± 3.5 22.0 (19.2–25.0)	22.5 ± 3.5 22.5 (20.0–25.0)	0.876*	−0.029 (−0.386, 0.329)
	Post-intervention	23.7 ± 3.4 24.0 (21.0–26.0)	22.7 ± 3.4 22.5 (20.0–25.0)	0.097*	0.305 (−0.055, 0.665)
	Two-month follow-up	23.5 ± 3.3 23.0 (21.0–25.7)	22.4 ± 3.5 22.0 (20.0–25.0)	0.079*	0.324 (−0.037, 0.683)
p-value (within-groups)		<0.001****	<0.007****		
Total	Baseline	159.1 ± 20.1 158.5 (146.2–174.7)	160.0 ± 20.4 159.5 (147.2–174.7)	0.809*	−0.044 (−0.402, 0.314)
	Post-intervention	179.8 ± 17.7 177.0 (169.0–192.7)	160.9 ± 19.5 159.5 (147.2–174.7)	<0.001*	1.017 (0.634, 1.395)
	Two-month follow-up	179.9 ± 17.2 178.5 (168.0–193.7)	160.1 ± 19.0 159.5 (148.2–174.0)	<0.001*	1.088 (0.702, 1.470)
p-value (within-groups)		0.001****	<0.018****		

* P: Independent samples t-test

** P: Mann–Whitney U test

*** P: Friedman test

**** P: Repeated Measures ANOVA

**Fig. 3:**
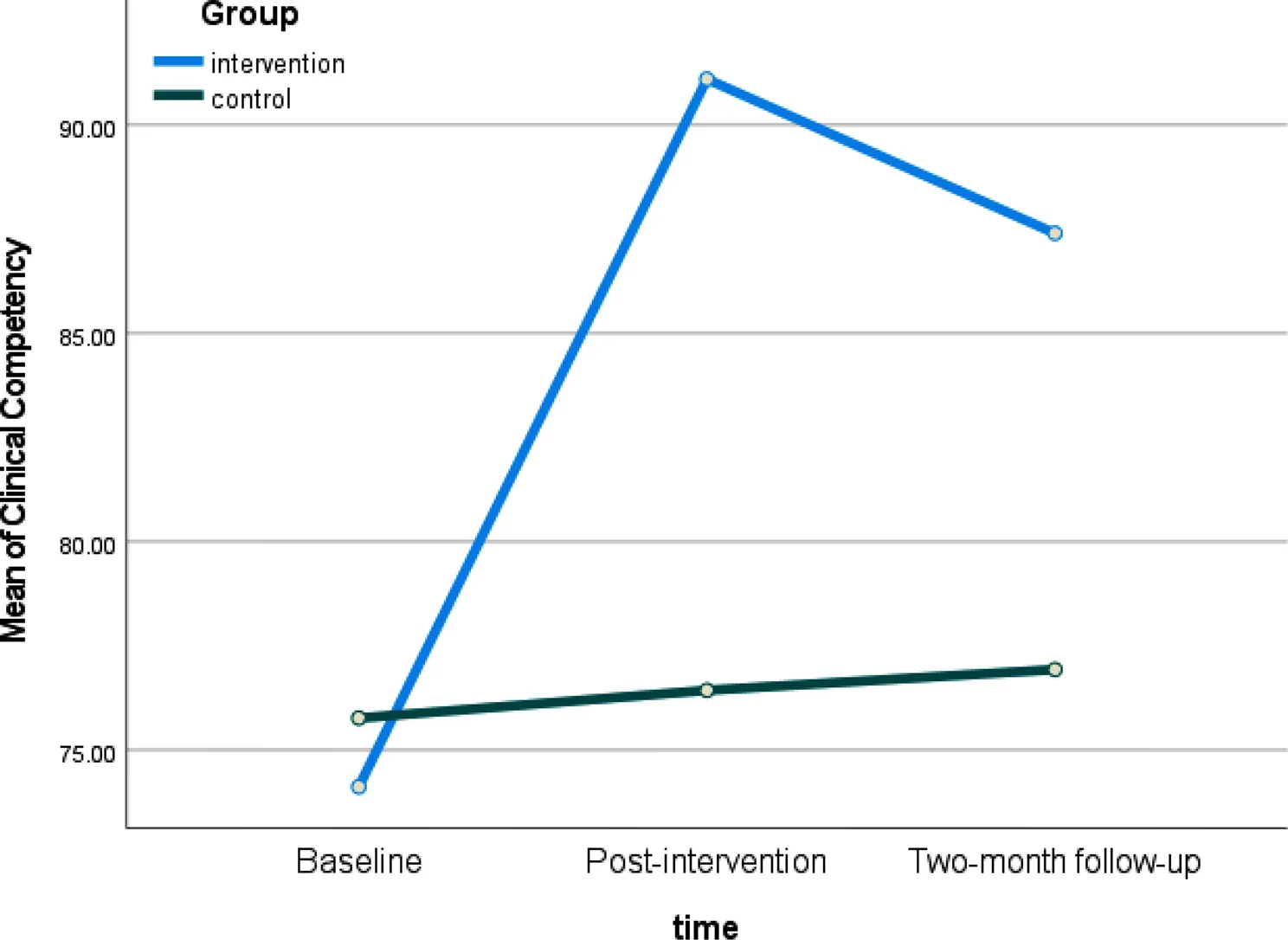
Mean clinical competence scores across three time points (baseline, post-intervention, and two-month follow-up) in the intervention and control groups

Because the assumption of sphericity was violated for several outcomes, Greenhouse–Geisser corrections were applied. Mixed repeated-measures ANOVA demonstrated significant main effects of time for all dimensions of clinical competence and for the total competence score (all *p* ≤ 0.001). Significant main effects of group were observed for clinical care, interpersonal relationships, legal–ethical practice, and total clinical competence, whereas no significant group effects were found for leadership, professional development, teaching–coaching, or research aptitude–critical thinking (*p* > 0.05). Importantly, significant time × group interaction effects were identified for all dimensions of clinical competence and for the total competence score (all *p* ≤ 0.001). These findings indicate that changes in clinical competence over time differed significantly between the intervention and control groups, with greater improvements observed among nurses who participated in the spiritual care education program (Table 6). The trends in clinical competence scores across the three assessment time points are presented in Fig. 3.

**Table 6:** Mixed repeated measures ANOVA results for clinical competence and its dimensions

Outcome	Effect	F (df)	*p*-value	Partial η^2^
Clinical Care	Time	259.5 (1.3, 159.5)	<0.001	0.687
	Group	32.9 (1,118)	<0.001	0.218
	Time × Group	252.7 (1.3, 159.5)	<0.001	0.682
Leadership	Time	53.6 (1.5, 183.2)	<0.001	0.312
	Group	0.9 (1,118)	0.350	0.007
	Time × Group	43.6 (1.5, 183.2)	<0.001	0.270
Interpersonal Relationships	Time	401.4 (1.3, 156.9)	<0.001	0.773
	Group	99.2 (1,118)	<0.001	0.457
	Time × Group	397.6 (1.3, 156.9)	<0.001	0.771
Legal–Ethical Practice	Time	111.3 (1.5, 179.7)	<0.001	0.485
	Group	4.1 (1,118)	0.044	0.034
	Time × Group	89.3 (1.5, 179.7)	<0.001	0.431
Professional Development	Time	77.1 (1.7, 198.7)	<0.001	0.395
	Group	3.6 (1,118)	0.059	0.030
	Time × Group	77.8 (1.7, 198.7)	<0.001	0.397
Teaching–Coaching	Time	88.9 (1.7, 197.4)	<0.001	0.430
	Group	2.8 (1,118)	0.097	0.023
	Time × Group	74.2 (1.7, 197.4)	<0.001	0.386
Research Aptitude–Critical Thinking	Time	50.2 (1.9, 222.5)	0.001	0.299
	Group	1.2 (1,118)	0.276	0.010
	Time × Group	35.7 (1.9, 222.5)	<0.001	0.232
Total Clinical Competence	Time	398.9 (1.4, 160.6)	<0.001	0.772
	Group	13.2 (1,118)	<0.001	0.100
	Time × Group	365.3 (1.4, 160.6)	<0.001	0.756

## Discussion

This randomized controlled trial was conducted between March 2024 and January 2025 with the aim of evaluating the efficacy of a structured spiritual care education program on nurses’ spiritual well-being and clinical competence. The primary hypothesis was that nurses participating in the intervention would demonstrate significantly greater improvements in both spiritual well-being and clinical competence compared with those in the control group receiving routine training.

The findings of this study supported this hypothesis. Compared with the control group, participants in the intervention group showed greater improvements across most study outcomes immediately after the intervention, and these improvements were largely maintained at the two-month follow-up assessment. Although minor fluctuations and, in some cases, small statistically significant changes were also observed in the control group over time, the magnitude of improvement was substantially greater in the intervention group. Furthermore, the significant time × group interaction effects and the observed effect sizes across most outcomes support the conclusion that the educational intervention contributed meaningfully to the observed changes beyond temporal effects alone. However, the relatively large magnitude of some effect sizes should be interpreted with caution, particularly given the self-reported nature of the outcome measures and the intensive, multi-component educational design of the intervention, which may amplify perceived changes.

The present study demonstrated that the spiritual care education program had a positive effect on nurses’ spiritual well-being. Although no significant between-group differences were observed at baseline, the intervention group showed marked improvements in total spiritual well-being as well as in both the religious and existential dimensions following the intervention, with these improvements persisting at the two-month follow-up. In contrast, the control group demonstrated either no significant changes or only small changes over time, with substantially smaller improvements compared with the intervention group. The significant interaction effects observed for all spiritual well-being outcomes further suggest that the pattern of change across time differed meaningfully between the two groups. Notably, the largest effects were observed in the existential dimension, indicating that the intervention may have had a particularly strong influence on nurses’ sense of meaning, purpose, and inner coherence in clinical practice. This is clinically meaningful, as existential well-being is more amenable to educational intervention than religious well-being, which is often rooted in stable personal beliefs [40], and has been linked to greater resilience and lower burnout among nurses [41, 42].

These findings are consistent with previous studies reporting beneficial effects of spiritual care education on nurses’ spiritual health and well-being. For example, Mehdipoorkorani and colleagues reported that a structured spiritual care program addressing multiple dimensions of spirituality significantly improved spiritual well-being among oncology nurses [13]. Similarly, Hu et al. found that a comprehensive training program incorporating reflective and experiential learning components improved nurses’ spiritual health [27]. While these findings are generally aligned with the present study, methodological limitations in some previous studies—including relatively small sample sizes, quasi-experimental designs, and limited follow-up periods—may reduce the strength and generalizability of their conclusions. In contrast, the randomized design and follow-up assessment employed in the present study provide additional evidence regarding the sustainability of intervention-related improvements over time. Furthermore, the persistence of improvements at two months in the present study suggests a durable internalized change rather than a transient effect, likely facilitated by the reflective structure of the program.

The sustained improvements observed in the intervention group may be related to the reflective and interactive structure of the educational program. In particular, the use of structured reflection based on the Gibbs reflective cycle, scenario-based discussions, and opportunities for shared clinical experiences may have facilitated deeper engagement with spiritual concepts and supported the integration of spiritual perspectives into professional practice. Consistent with the theoretical foundations of reflective learning, this structured approach moves participants beyond description toward analysis and action planning, transforming abstract knowledge into personally meaningful insights [43]. These educational strategies may also have enhanced participants’ self-awareness and sensitivity toward the spiritual dimensions of patient care, thereby contributing to improvements in spiritual well-being [9].

The findings of the present study also demonstrated that the spiritual care education program had a significant positive effect on nurses’ clinical competence. Although no significant between-group differences were observed at baseline, participants in the intervention group demonstrated significantly higher scores in total clinical competence and in several competence dimensions following the intervention, including clinical care, interpersonal relationships, legal–ethical practice, professional development, and teaching–coaching. These improvements remained evident at the two-month follow-up assessment. In contrast, the control group demonstrated either minimal or less pronounced changes over time. Effect size analyses further indicated that the magnitude of the intervention effect ranged from small to very large, with particularly strong effects observed in the domains of clinical care and interpersonal relationships. This finding likely reflects the inherent nature of spiritual care education, which fundamentally emphasizes therapeutic presence, empathetic communication, and compassionate connection—competencies that are central to both interpersonal relationships and holistic clinical care. By reframing clinical encounters as opportunities for humanistic engagement rather than task completion, the program appears to have directly strengthened these core nursing competencies. This is consistent with previous evidence showing that spiritual care education enhances communication skills and patient-centered care by targeting the relational dimensions of nursing practice [44].

These findings are broadly consistent with previous studies suggesting that spiritual care education may strengthen multiple aspects of nursing competence. For example, Abusafia et al. reported that participation in a structured spiritual care training module improved overall nursing competence following a short-term intervention [26]. Similarly, Hsieh et al. found that a scenario-based spiritual care course enhanced competence in spiritual care practice among clinical nurses [12]. However, important methodological differences should be considered when comparing findings across studies, including differences in intervention duration, educational methods, follow-up periods, and outcome measures. Compared with several earlier studies, the present study provides additional evidence regarding the persistence of intervention-related improvements beyond the immediate post-intervention period. The sustained or even enhanced improvements observed at the two-month follow-up may be explained by the nature of clinical competence development. Unlike knowledge that can be acquired through passive learning, clinical competence requires deliberate practice and application in real-world settings [45]. The interval between the post-intervention and follow-up assessments likely provided participants with opportunities to apply their newly acquired skills in clinical practice, thereby reinforcing and consolidating their learning. This is consistent with skill acquisition theories, which emphasize that competence develops progressively through cycles of practice, feedback, and reflection [46].

Interestingly, no significant between-group differences were observed for the leadership and research aptitude–critical thinking dimensions at the post-intervention or follow-up assessments. Nevertheless, significant time × group interaction effects were identified for these domains in the repeated-measures analyses, suggesting that the trajectory of change over time differed between groups even though between-group differences at specific assessment points did not consistently reach statistical significance. This may be explained by the fact that the spiritual care education program was specifically designed to enhance competencies directly related to patient-centered and holistic care—such as clinical care and interpersonal relationships—rather than higher-order competencies like leadership or research aptitude. These findings may indicate that leadership and research-related competencies are less responsive to short-term educational interventions and may require more targeted interventions, organizational support, or practice-based reinforcement to achieve measurable improvement [47].

Although the findings of the present study are generally consistent with the growing body of evidence supporting spiritual care education, some studies have reported more heterogeneous or limited effects across outcome domains. For instance, Vlasblom et al. found that while spiritual care training improved nurses’ attitudes and patients’ perceptions of support, improvements were not consistently observed across all practice-related outcomes [48]. Such variability may be influenced by contextual factors including organizational culture, baseline competence levels, intervention intensity, and duration of follow-up. Therefore, differences across studies should be interpreted within the context of methodological and clinical variations.

### Strengths and limitations

This study has several strengths. First, unlike many previous studies that have focused on either spiritual well-being or clinical competence alone, the present study simultaneously evaluated both outcomes, providing a more comprehensive understanding of the effects of spiritual care education. Second, the inclusion of repeated assessments at baseline, immediately post-intervention, and two-month follow-up enabled evaluation of both immediate and sustained short-term effects of the intervention. Third, the randomized controlled design and the baseline comparability between groups strengthened the internal validity of the findings. Despite these strengths, several limitations should be acknowledged. The study was conducted in a single teaching hospital, which may limit the generalizability of the findings to other healthcare settings. In addition, all participants were female nurses, potentially restricting the applicability of the results to more diverse nursing populations. The study outcomes were assessed using self-reported instruments rather than objective or observational measures, which may have introduced response bias and may not fully capture actual clinical performance. Furthermore, although participants were randomly allocated before outcome assessment, the sequential completion of assessments may have introduced temporal bias due to possible changes in external conditions over time. In addition, the trial was registered retrospectively due to administrative delays, which may limit full transparency despite adherence to the predefined protocol. Finally, although the two-month follow-up provided evidence of short-term retention of the intervention effects, longer follow-up periods are needed to determine the long-term sustainability of these improvements.

## Conclusion

The findings of this randomized controlled trial demonstrated that the spiritual care education program significantly enhanced nurses’ spiritual well-being and clinical competence. Improvements were observed immediately after the intervention and were maintained at the two-month follow-up, indicating sustained short-term benefits. The intervention was associated with improvements in both religious and existential well-being, as well as multiple domains of clinical competence, particularly clinical care, interpersonal relationships, and legal–ethical practice. These findings suggest that structured and scenario-based spiritual care education may serve as an effective approach for strengthening nurses’ holistic competencies and supporting professional practice. Incorporating spiritual care education into continuing education and in-service nursing programs may help promote more comprehensive and patient-centered care. Future research is recommended to evaluate the long-term sustainability of these effects and to examine the applicability of similar interventions across different healthcare settings and populations.

## Data Availability

The datasets generated and/or analyzed during the current study are available from the corresponding author upon reasonable request.

## Abbreviations

SPSS: Statistical Package for the Social Sciences
CIRN: Clinical Competence Questionnaire for Registered Nurses
SWBS: Spiritual Well-Being Scale
